# Deep Transfer Learning Approach for Localization of Damage Area in Composite Laminates Using Acoustic Emission Signal

**DOI:** 10.3390/polym15061520

**Published:** 2023-03-19

**Authors:** Jingyu Zhao, Weihua Xie, Dong Yu, Qiang Yang, Songhe Meng, Qihui Lyu

**Affiliations:** 1 Science and Technology on Advanced Composites in Special Environment Laboratory, Harbin Institute of Technology, Harbin 150080, China; 2 School of Science, Harbin Institute of Technology, Shenzhen 518055, China

**Keywords:** composites, damage localization, acoustic emission, deep transfer learning

## Abstract

Intelligent composite structures with self-aware functions are preferable for future aircrafts. The real-time location of damaged areas of composites is a key step. In this study, deep transfer learning was used to achieve the real-time location of damaged areas. The sensor network obtained acoustic emission signals from different damaged areas of the aluminum alloy plate. The acoustic emission time-domain signal is transformed into the input image by continuous wavelet transform. The convolutional neural network-based model automatically localized the damaged area by extracting features from the input image. A small amount of composite acoustic emission data was used to fine-tune some network parameters of the basic model through transfer learning. This enabled the model to classify the damaged area of composites. The accuracy of the transfer learning model trained with 900 samples is 96.38%, which is comparable to the accuracy of the model trained directly with 1800 samples; the training time of the former is only 17.68% of that of the latter. The proposed method can be easily adapted to new composite structures using transfer learning and a small dataset, providing a new idea for structural health monitoring.

## 1. Introduction

Fiber-reinforced composites have been widely used in the marine, automobile, and aircraft industries due to their excellent mechanical properties [1,2]. However, the influence of various external loading conditions [3,4,5,6,7], such as low-velocity impacts, can significantly affect the load-bearing capacity of composites, thus seriously compromising the safety of the structural system. Many studies have used non-destructive testing methods such as thermal imaging [8] and ultrasonic C-scanning [9] to locate and evaluate invisible damage in composite structures. However, these methods require specialized equipment and cannot quickly locate the damaged area. Therefore, it is urgent to develop an efficient and real-time structural health monitoring (SHM) system to evaluate the damaged area of composite structures.

Acoustic emission (AE) is a technique that can extract and characterize material damage characteristics in real time. Elastic waves generated during material damage are detected by AE sensors and converted into electrical signals. Therefore, AE technology can monitor the condition of the structure without external excitation [10]. Many researchers have proposed AE-based damage localization methods for composite structures [11,12,13,14]. Most of these studies require the extraction of specific characteristics of the AE signal, such as the time of arrival (TOA). The damaged area is determined according to the location of the sensor and the propagation velocity of the stress wave in the material. The method assumes that the wave velocity is constant in all directions. However, due to the strong anisotropy of the composite, the wave velocity is different in different directions. The wave propagation is also affected by the interface and defects in the composites. In addition, different threshold values will result in different TOA [15]. Therefore, the application of the TOA method is limited.

In recent years, with the development of data-driven methods, machine learning (ML) methods represented by deep learning (DL) have been applied to damage/defect detection and structural performance evaluation. Sause et al. [16] used neural networks to learn the AE signals of fiber-reinforced composites to predict the failure load. Patel et al. [17] predicted the effect of microstructure on the crack initiation of fiber-reinforced ceramic composites by the ML model. Califano et al. [18] developed an SHM method based on a neural network to detect whether there is damage in the carbon fiber/epoxy composite panel. Santosh Kumar et al. [19] studied the relationship between specific wear rate and mechanical properties of glass fiber-reinforced epoxy composites through three ML algorithms. Ramasamy et al. [20] used an artificial neural network (ANN) to predict impact damage tolerance based on AE data obtained during online monitoring of low-velocity impact tests. Sharif-Khodaei [21] developed an ANN-based model that can estimate the location of the impact by using sensor signal data. Datta [22] proposed a localization impact model based on least squares support vector regression and applied it to a carbon/epoxy composite plate structure. Kundu [23] used metamodeling techniques to learn the mapping relationship between AE signal characteristics (spatial and temporal) and damage properties, and a hierarchical Bayesian inference framework was used to localize and characterize the damage during the online monitoring phase. However, different pre-defined impairment features may lead to different recognition results. To avoid this effect, a DL-based image classification technique was used [24,25]. The image classification technique represented by a convolutional neural network (CNN) is a deep learning architecture that has been widely studied in recent years [26]. Khan [27] used CNN models and vibration data to identify delamination damage in composites. Tao [28] used deep learning algorithms and ultrasound to characterize fatigue damage in composite laminates. Atha [29] proposed a method to detect corrosion defects on a metal surface based on 2D CNN. Kumar [30] used a deep learning algorithm to classify defects in sewer inspection images automatically. The advantage of CNN is that it can process image input and generate similar feature values from local areas with similar patterns. However, CNN requires a large amount of data to train the model parameters, and the lack of data can lead to non-convergence or over-fitting of the model. In the field of composites, the high cost of experiments and numerical simulation makes it difficult to obtain a large amount of data.

Using a small amount of data to obtain a model with good performance has become a pressing problem for DL-based composite structural health assessment. The damaged area location method proposed in this study has the following advantages: (1) Using the transfer learning strategy, the damaged area location model can be transferred to different structures with a small amount of data, which greatly reduces the cost of building the composite material database. (2) The trained model can be used as an intelligent perception module for vehicles with the composite structure to locate the damaged area in real time. In addition, related studies have shown that reducing the complexity of data sets can potentially improve the accuracy of deep learning models [31,32]. In this paper, we reduce the complexity of the data set by transforming the original time–domain data. Then, the DL model is trained with low-cost AE time–frequency data of aluminum alloy, and then the damage location (source domain) of the aluminum alloy sheet is transferred to the damage location of fiber-reinforced composites (target domain) by transfer learning. The similarity of acoustic emission signal propagation in solid materials provides a basis for the mobility of damaged locations of different materials.

The contents of this paper are organized as follows: In Section 2, a series of pencil lead break experiments in different areas were carried out on aluminum alloy plates and carbon-fiber-reinforced plastics (CFRP) laminates to simulate material damage, and the AE signals are recorded using a network of sensors. In Section 3, the AE time-domain signal is converted into time–frequency scale diagrams by continuous wavelet transform to generate datasets for training and testing the DL model. At the same time, a transfer framework is proposed to transfer the damage location model of the aluminum alloy plate to the damage location of CFRP laminate. In Section 4, the effect of transfer learning on the performance of the DL model is compared.

## 2. Materials and Methods

### 2.1. Material Preparation

**Figure 1:**
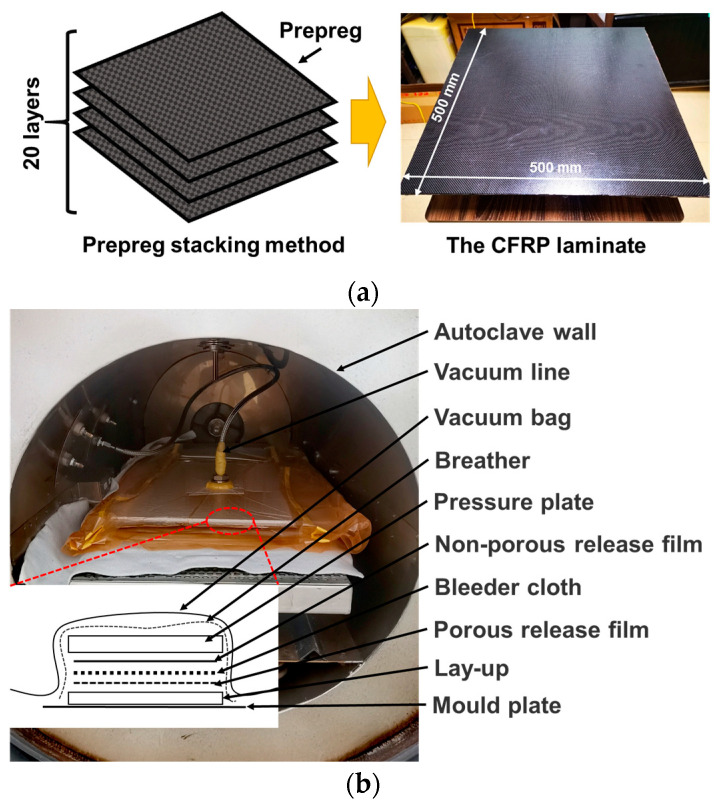

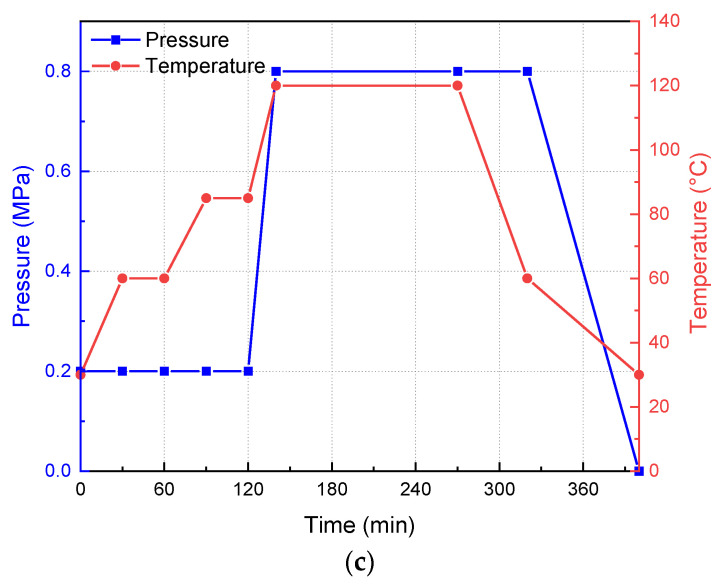
(**a**) Fabrication process; (**b**) manufacturing process for CFRP composite laminates; (**c**) hot-pressing process parameters.

Aluminum alloy plates and composite laminates of the same size were prepared for AE experiments. The grade of the aluminum alloy plate is 2A12T4 and the size is 500 × 500 × 5 mm. The CFRP composite laminates were prepared by hot pressing plain weave prepregs. The prepreg consists of T300 carbon fiber fabric and epoxy resin (approximately 42% resin volume fraction), with a thickness of approximately 0.25 mm. The prepreg was cut to a predetermined size (500 × 500 mm) and stacked 20 layers at a lay-up angle of 0°, as shown in Figure 1a. The laminate preparation process is shown in Figure 1b. The prepreg is laid on the surface of the mold plate with a release agent, then covered with a bleeder cloth and a non-porous release film. These are sealed in a vacuum bag and placed in an autoclave. Before heating and curing, the vacuum bag is vacuumed to remove air and volatiles. The curing process of the laminate is shown in Figure 1c. After heating to 120 °C, a pressure of 0.8 MPa is applied to the prepreg to ensure that air and volatiles are removed without extruding too much resin. The laminate begins to cool after 2–3 h of heat and pressure. For the cooling rate, it has been shown that a lower cooling rate is more conducive to crystallization [33], which means that better mechanical properties can be obtained. Therefore, the composite laminates in this study were cooled naturally in an air environment.

### 2.2. Damage Area Location Experiment Based on Acoustic Emission

**Figure 2:**
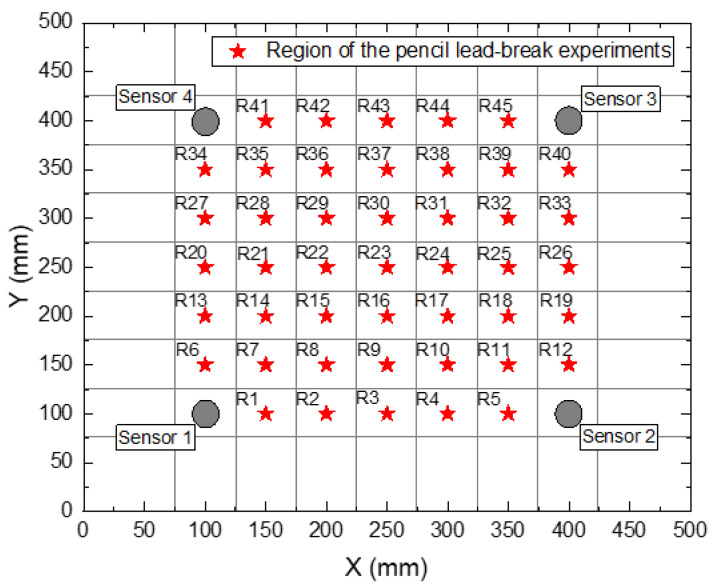
Schematic illustration of the area of the pencil lead break on the aluminum alloy plate/composite laminate.

Researchers have used various types of artificial damage sources (pencil lead break, impact, fatigue) as AE signal sources [34,35]. The AE source represented by the Hsu–Nielsen pencil lead break (PLB) is a commonly used artificial AE source with good experimental repeatability [34]. In this study, the AE source generated by the PLB experiment was used to simulate the AE signal caused by material damage. The PLB signal source is generated by pressing a pencil core (0.5 mm diameter, 5 mm long, at a 45-degree angle to the panel) onto the top surface of the panel and breaking it approximately 3 mm from the tip. Note that the difference in lead core diameter and break length affects the AE signal. In the experiment, the angle, diameter, and length of the lead core fracture were kept as consistent as possible to reduce this effect. To distinguish the reflected wave from the recorded waveform, the distance between the sensor and the panel boundary is set at 75 mm, as shown in Figure 2. The remaining area of the panel was divided into 49 square areas with sides of 50 mm. AE sensors were placed in the 4 corners and marked as sensors 1 to 4. The remaining 45 areas were used as damaged areas, and AE events caused by PLB in these areas were recorded using a sensor network. The damaged area is uniformly expressed as R + number, e.g., area 1 is recorded as R1. To build the database, the PLB experiment was randomly repeated 40 times in each area, and 1800 impacts were recorded in 45 areas. Even within the same area, the location of each random impact is different, resulting in a highly variable database.

**Figure 3:**
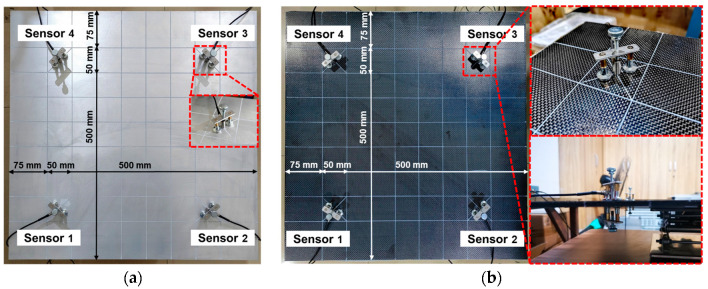
Experimental setup for the area location of acoustic emission sources: (**a**) aluminum alloy plate; (**b**) CFRP composite laminates.

The panels are supported by polymer foam to minimize vibration and energy transfer from the environment to the panels. A cylindrical piezoelectric AE sensor (Figure 3) with a diameter of 5 mm was used in the experiment to receive the AE signal propagating in the panel. To maintain good contact between the panel surface and the sensor, vacuum silicone grease is used as an acoustic coupling agent between the panel surface and the AE sensor. The data acquisition device is a DS5 series AE system (Beijing Softland Times Scientific &Technology Ltd., Beijing, China). The main parameters were set as follows: the pre-amplification gain of the AE sensor was set to 40 dB, the trigger threshold was set to 10 dB, and the sampling frequency was set to 3 MHz.

### 2.3. CNN-Based Damage Area Localization Method

In this section, a CNN-based damage area localization model is developed. The powerful feature extraction capability of CNN is applied to learn the nonlinear mapping relationship between CWT images and damage areas to achieve damage location identification. For small training datasets, a transfer learning framework is developed to improve the generalization performance of the model.

#### 2.3.1. Dataset Generation

In order to train the deep learning model, the captured AE signals must be processed in the time–frequency domain. Based on the AE signals obtained by PLB experiments on aluminum alloy plates and composite laminates in Section 2.2, the damage area location datasets of the two materials are constructed by using continuous wavelet transform technology.

Continuous wavelet transform (CWT) is a time–frequency transform method, which is widely used in SHM applications [36,37]. The characteristic of CWT is that the signal can be analyzed at multiple resolutions (wavelet has different resolutions in different frequency segments), which makes CWT an ideal method for analyzing non-stationary signals such as PLB.

**Figure 4:**
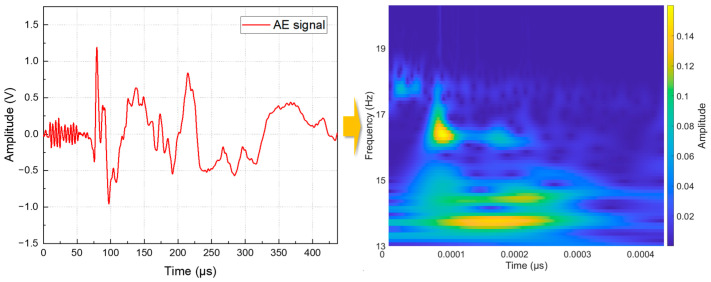
The time-domain signal of a typical AE event and the corresponding time–frequency scale diagram.

CWT C(τ,f) is defined as follows:

(1) $$C\left(\tau ,f\right)=\frac{1}{\sqrt{s\left(f\right)}}\int_{-\infty }^{+\infty }x\left(t\right)\psi^{*}\left(\frac{t-\tau }{s\left(f\right)}\right)dt$$

where x(t) is the acquired signal, $\psi^{*}$ is the complex conjugate of the mother wavelet ψ(t), f is the frequency, τ is the translational parameter, the non-dimensional scale parameter s is defined as $s\left(f\right)=f_{c}f_{s}/f$, $f_{c}$ is the central frequency, and $f_{s}$ is the sampling frequency. In this study, the Morse wavelet is adopted as the mother wavelet, defined as

(2) $$\psi (t)=\frac{1}{\sqrt{\pi f_{b}}}\exp\left(2\pi f_{c}jt-\frac{t^{2}}{f_{b}}\right)$$

where $f_{b}$ is the bandwidth. To avoid the interference of the signal intensity on the location of the damaged area, the AE signal data measured by the AE sensor were normalized to the interval (0, 1). Substitute the original AE time domain signal into Formula (1)—that is, perform a convolution operation between the original signal and the parent wavelet to obtain a two-dimensional matrix, where one dimension is time and the other dimension is frequency. Convert this matrix into an RGB image to obtain the time–frequency scale diagram of the original signal. Figure 4 shows the time-domain signal of a typical AE event and the corresponding time–frequency scale diagram. In the time–frequency scale diagram, the abscissa represents time, the ordinate represents frequency, and the color represents amplitude, indicating the damage area information.

**Figure 5:**
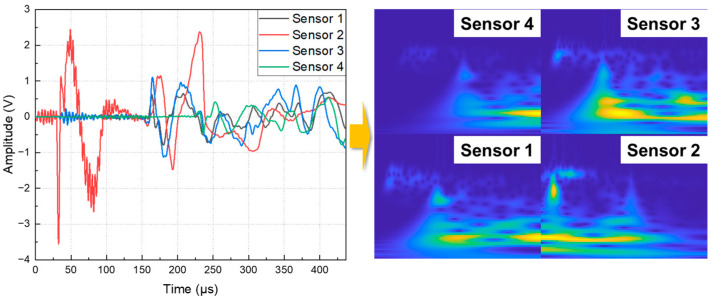
The construction process of the input image of the deep learning model.

The time–frequency scale diagrams of the data from the four sensors are arranged according to the position of the sensors, and the obtained RGB images are used as the input data of the deep learning model, as shown in Figure 5. The CWT image converted from the acoustic emission signal of the pencil lead break contains the location and time information of the damaged source. The dataset includes 1800 samples and is divided into the training set, validation set, and test set according to the ratio of 60:20:20. The training set is used to learn model parameters. The validation set is used to adjust the hyperparameters of the model. The test set is used to evaluate the generality of the model.

#### 2.3.2. Convolutional Neural Networks

Convolutional neural networks are a class of deep neural networks most commonly used in visual image recognition. CNN mainly consists of convolutional layers, pooling layers, and fully connected layers. 

The convolutional layer performs convolutional operations on the input data to extract feature mappings, whereas the shallow convolutional layer extracts basic features, such as edges and contours, and the deep convolutional layer extracts abstract features, such as the entire image. The convolutional layer consists of a set of filters (convolutional kernels) with learnable weights that perform the main computational tasks in CNN. In a 2D convolutional layer, forward propagation can be expressed as:

(3) $$g_{i}=f\left[\sum_{n=1}^{N}Conv2D\left(w_{i,n},a_{n}\right)+b_{i}\right]$$

where $g_{i}$ is the computed result of the i−th convolution kernel, $a_{n}$ is the input data of size $N_{a}\times 1\times N$, $w_{i,n}$ is the weight matrix of the i−th convolution kernel of size $N_{w}\times 1\times N$, $b_{i}$ is the deviation of the i−th convolution kernel, and f is the activation function. The advantage of the activation function is that it introduces nonlinearity into the CNN, which is beneficial for detecting the nonlinear features of the data. The common activation functions are sigmoid, ReLU, and tanh. In this study, the ReLU function is used, which is more robust to various disturbances and avoids the gradient disappearance problem to some extent.

The pooling layer achieves data reduction by down-sampling. The pooling layer is typically located between two convolutional layers and serves as a regularizer. It reduces both the amount of data passed to the next layer and the amount of computation required. Although the pooling layer loses data information, it prevents overfitting. Max pooling is the commonly used form of pooling and is expressed as:

(4) $$p_{i}\left(j\right)=\max_{\left(j-1\right)\times m\leq k\leq j\times m}\left(a_{i}\left(k\right)\right)$$

where $a_{i}\left(k\right)$ is the i−th feature map input to the k−th cell of the pooling layer, and $p_{i}\left(j\right)$ is the output from the i−th feature map of the j−th cell of the pooling layer. The size of the pooling layer filter is m×1. The fully connected layer classifies the feature mappings extracted from the convolutional and pooling layers.

**Figure 6:**
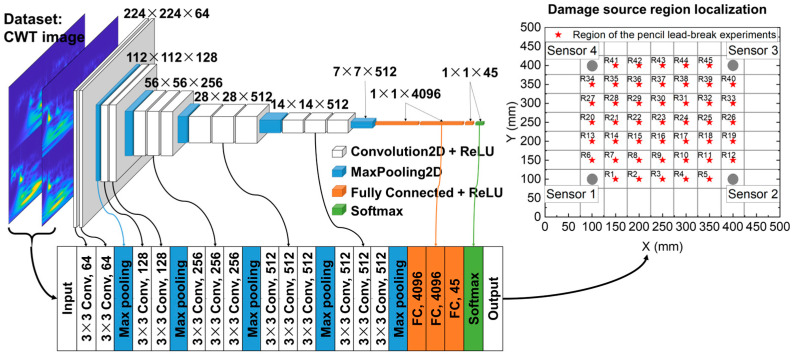
A deep learning-based framework for locating damaged areas.

In this study, the VGG16 architecture is used as the damage area detection model. VGG16 is a 2D CNN architecture proposed by Simonyan [38]. Its outstanding contribution is to prove that it can effectively improve performance through very small convolution and increase network depth. The detailed configuration of the network structure is shown in Figure 6, including 13 convolutional layers, 5 pooling layers, and 3 fully connected layers, and excluding the activation layer. The model optimizer is Adam. The effects of different mini-batch sizes (32, 64, and 128) and initial learning rates (0.001, 0.01, and 0.1) on the model convergence and accuracy are compared. Finally, the initial learning rate was set to 0.001, and the mini-batch size used for each training iteration was set to 32.

#### 2.3.3. Transfer Learning Framework

A deep learning model typically contains millions of parameters, so a considerable amount of data is needed to effectively train all the parameters to achieve highly accurate predictions. However, in many cases, it is difficult to obtain a large amount of data, which limits the application of deep learning models. A small dataset will also greatly affect the generalization ability of the model. The solution to overcome these problems is called transfer learning [39], in which the knowledge learned by the model in one domain is applied to other domains. Typically, pre-trained models that have been trained on large datasets are used to assist in learning new tasks. These pre-trained models can extract shallow basic features and deep abstract features. Specifically, some of the network parameters of the pre-trained model are frozen to retain its ability to extract shallow features, while the parameters of the other part of the network are retrained using data from the new domain to adjust its higher-order feature representation to make it more relevant to the specific task [40]. 

**Figure 7:**
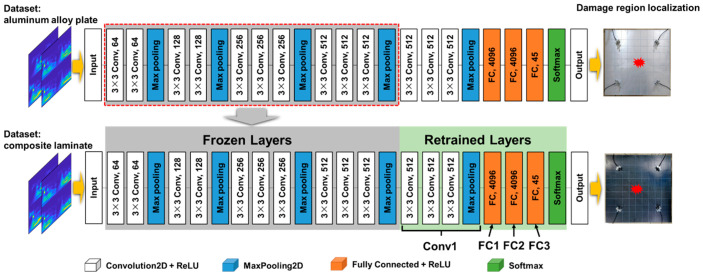
A transfer learning framework for locating damaged areas.

Acoustic emission refers to the phenomenon in which a material locally emits transient elastic waves due to the rapid release of energy. Acoustic emission is also called stress wave emission. The elastic wave emitted by the acoustic emission source propagates through the solid medium to the surface of the object, causing the surface to vibrate mechanically. In solid media, the reflection and refraction of stress waves follow the same physical laws. In addition, due to the difference in the elastic modulus, density, and internal structures of different media, the propagation speed and attenuation of the stress wave are also different. The similar propagation laws and different propagation details of stress waves in different solid media support the rationality and effectiveness of transfer learning. In this study, the VGG16 model is first pre-trained using the AE data of the aluminum alloy plate to obtain the basic model of transfer learning. Next, one part of the network parameters of the pre-trained model is frozen to retain its ability to extract the shallow features of time–frequency image data, while the other part of the network parameters is retrained using the AE data of CFRP laminates to adjust its higher-order feature representation and make it more relevant to the damage area identification task of composite laminates, as shown in Figure 7. The performance of the model under different transfer strategies will be illustrated and discussed in Section 3.

## 3. Results and Discussion

### 3.1. Pre-Trained Model Training and Testing

**Figure 8:**
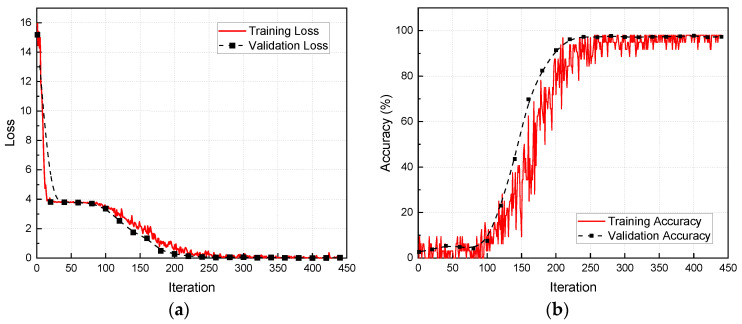
(**a**) Model training loss and validation loss; (**b**) model training accuracy and validation accuracy.

The DL model is trained and tested based on the AE dataset of the aluminum alloy plate to obtain a pre-trained model for composite laminate. First, the hyperparameters of the DL model are initialized and the training set is used to train the model parameters. Second, the validation set is used to verify the prediction accuracy of the model, and the hyperparameters of the model are adjusted according to the inference results of the validation set. Finally, when the prediction accuracy meets the requirements, the training is stopped and the model is saved. After 440 iterations, both the loss and accuracy of the model converge, and the accuracy of the model on the validation set is 97.3%, as shown in Figure 8. Based on the personal computer of this study (CPU Intel(R) Core(TM) i7-12700 2.10 GHz) and the size of the dataset, it takes about 3.3 h for the model to complete convergence training.

**Figure 9:**
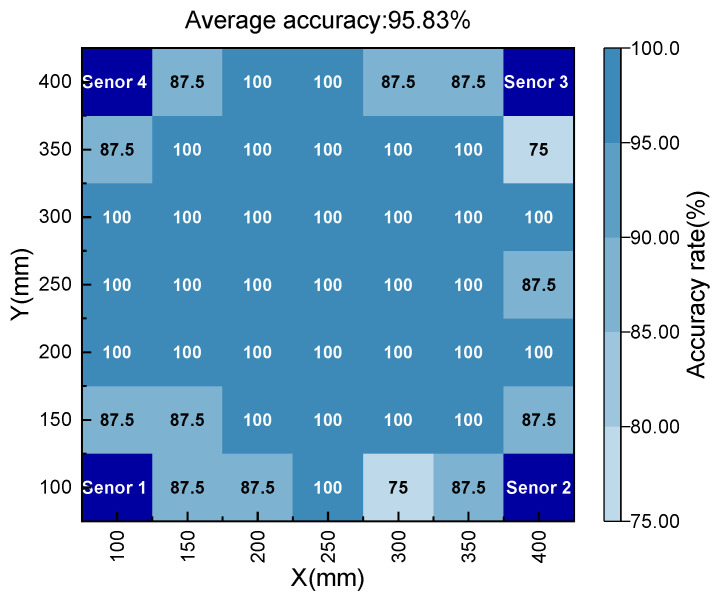
Identification accuracy of DL model on aluminum alloy test set.

**Figure 10:**
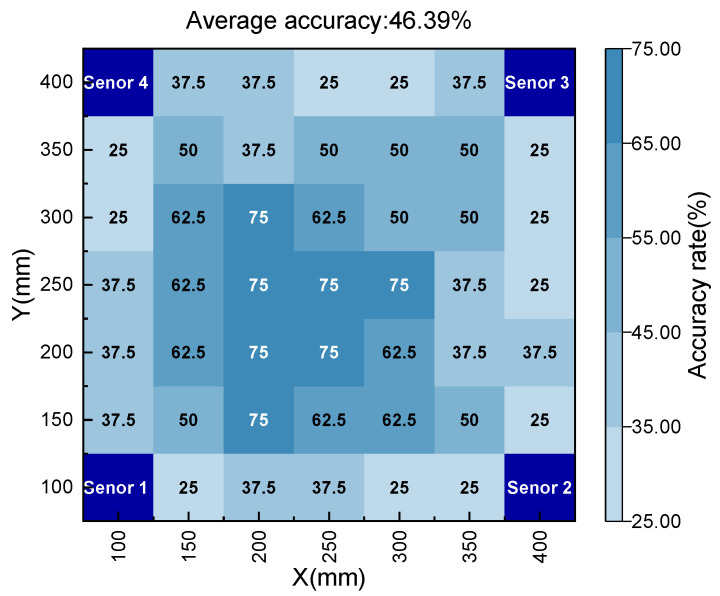
Identification accuracy of DL model on CFRP laminates test set.

The performance of the model on the test set is shown in Figure 9, and the average recognition accuracy of the damaged area is 95.83%. Moreover, it can be seen that the model has a higher prediction accuracy for the damaged area distributed in the center of the plate, but a lower prediction accuracy for the damaged area distributed at the edge. There are two reasons for this result: (1) the uncertainty of the experimental setup, and (2) the dispersion and edge reflection during elastic wave propagation. In addition, 20% of the composite laminate AE dataset was directly input into the pre-training model, and the damage area identification results are shown in Figure 10; the average recognition accuracy of the damaged area is 46.39%. The accuracy, precision, and recall indicators of the two test sets are shown in Table 1. It can be seen that the DL model trained by aluminum alloy data can still maintain a certain accuracy on composite material data.

### 3.2. Damage Area Localization Based on Transfer Learning

**Figure 11:**
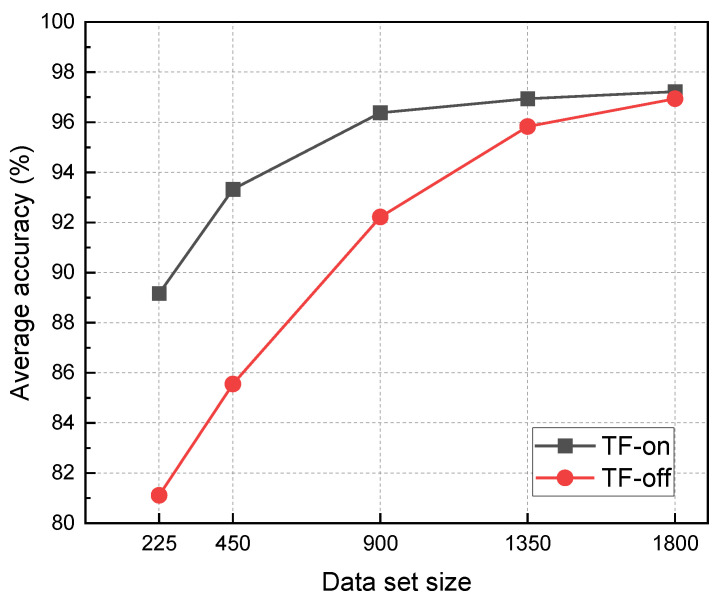
The influence of transfer learning on the accuracy of damage area identification.

According to the transfer learning framework shown in Figure 7, the transfer learning from the damaged area location of the aluminum alloy plate to the damaged area location of composite laminate is realized. First, the CNN model is trained with the AE data set of aluminum alloy plate to obtain the pre-training model. Then, the model parameters of the first few layers of the pre-training model are frozen, and the composite laminate AE data set is used to retrain the last three fully connected layers (FC1, FC2, FC3) and the last convolution block (Conv5, including three Convolution2D layers + ReLU layers + MaxPooling2D layers) of the pre-training model. The recognition accuracy of the model with and without the transfer learning framework is shown in Figure 11. Adding more samples to the training dataset helps the DL model learn and understand the data better, allowing it to make more accurate predictions. The accuracy of the damaged area increases for both models as the number of samples increases.

**Figure 12:**
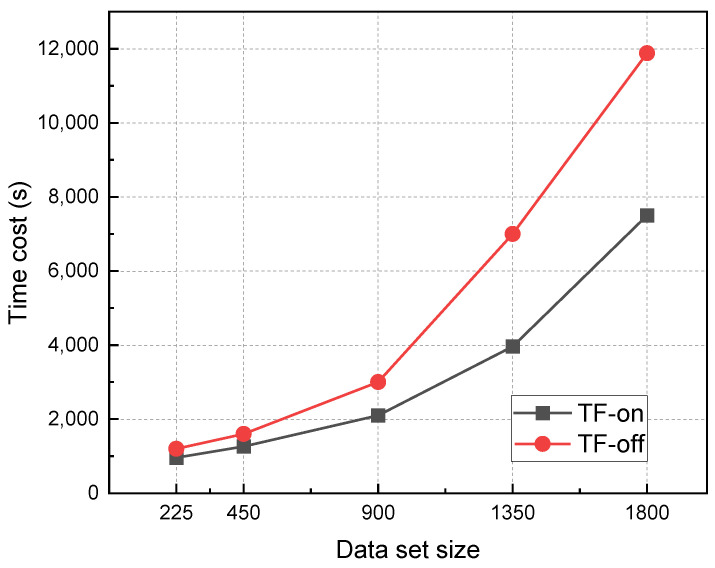
The influence of transfer learning on training efficiency.

However, the training cost of the transfer learning model is significantly lower than that of the direct training model. In terms of the amount of data required to train the model, the accuracy rate (96.38%) of the transfer learning model trained with 900 training samples is already comparable to that of the direct training model (96.94%) trained with 1800 samples. This shows that transfer learning can achieve excellent model performance with a small amount of data. When the sample size reaches 1350, the accuracy of the transfer learning model has stabilized at 96.94%, while the accuracy of the direct training model is still rising, which indicates that the sample size of 1350 is not enough for the direct training model. In addition, the accuracy obtained by the TOA method is 73.89%, which is significantly lower than that of the deep learning model. In contrast, in terms of model training time, when the accuracy rate is similar, the training time of the transfer learning model (accuracy: 96.38%, dataset size: 900) is only 17.68% of that of the direct training model (accuracy: 96.94%, dataset size: 1800), as shown in Figure 12.

**Figure 13:**
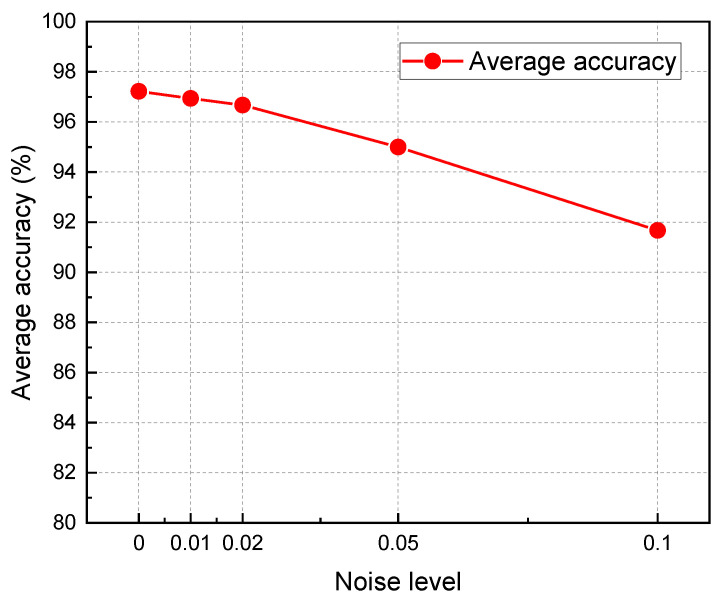
Recognition accuracy under different noise levels.

In order to verify the robustness of the transfer learning model, different levels of gaussian noise are added to the time–frequency images in the test set, which are input into the transfer model trained by 1800 samples. As shown in Figure 13, the influence of different noise levels on the recognition accuracy of the model is illustrated. When the noise variance reaches 0.05, the accuracy of the model only decreases from 97.22% to 95%, indicating that the model has good robustness.

## 4. Conclusions

This study proposes a method based on deep CNN and transfer learning for damage area real-time localization of CFRP composite laminates. The deep transfer learning model is well able to identify the location of damaged areas in composite laminates in real time. Instead of manually extracting the discriminative features of the AE signal, this DL-based model can directly localize the damaged area of composite laminates by applying CWT to the original acoustic emission signal. By fine-tuning some parameters of the pre-trained model, the number of model parameters and samples that need to be updated is greatly reduced and the training efficiency of the model is improved. When the training sample size is 900, the prediction accuracy of the transfer learning model is equivalent to that of the model trained directly with 1800 samples. At the same time, the transfer learning model can maintain high recognition accuracy under certain noise and has good robustness. The wrong identification of damage area locations usually occurs near the edge of laminates and sensors. There are two reasons for this result: (1) the uncertainty of the experimental setup, and (2) dispersion and edge reflection during elastic wave propagation. The present study demonstrated that the proposed damage area localization method can be easily adapted to new composite structures and damage types using transfer learning and small sample sizes. This study will provide a new idea for real-time structural health monitoring and the intelligent perception of composite materials.

Due to the high cost of composite experiments, the acoustic emission signal from the damaged source can only be simulated by non-destructive experiments, such as the Hsu–Nielsen pencil lead break experiment used in this study. However, this is still different from real composite damage. To overcome these limitations, two future research plans have been established: (1) create a finite element model to simulate the propagation of stress waves caused by impact and acoustic emission signals generated during composite damage, which is desirable to locate the damaged area more efficiently; (2) use deep learning algorithms to identify the relationship between acoustic emission signals and failure modes of composites.

## Figures and Tables

**Table 1:** The accuracy, precision, and recall indicators of the two test sets.

Test Set	Accuracy	Precision	Recall
Aluminum alloy	95.83%	95.83%	96.08%
CFRP laminate	46.39%	45.56%	46.48%

## Data Availability

Data is contained within the article.
